# Peripheral blood correlates of virologic relapse after Sofosbuvir and Ribavirin treatment of Genotype-1 HCV infection

**DOI:** 10.1186/s12879-020-05657-5

**Published:** 2020-12-04

**Authors:** Cody Orr, Wenjie Xu, Henry Masur, Shyam Kottilil, Eric G. Meissner

**Affiliations:** 1 Division of Infectious Diseases, Medical University of South Carolina, 135 Rutledge Ave, MSC752, Charleston, SC 29425 USA; 2 Nanostring Technologies, Seattle, WA USA; 3 Critical Care Medicine Department, NIH Clinical Center, National Institutes of Health, Bethesda, MD USA; 4 Division of Clinical Care and Research, Institute of Human Virology, University of Maryland School of Medicine, Baltimore, MD USA; 5 Department of Microbiology and Immunology, Medical University of South Carolina, 135 Rutledge Ave, MSC752, Charleston, SC 29425 USA

**Keywords:** Hepatitis C virus, Direct acting antiviral, Gene expression analysis, Sustained virologic response, Relapse

## Abstract

**Background:**

Treatment of chronic hepatitis C virus infection with direct acting antiviral therapy results in viral elimination in over 90% of cases. The duration of treatment required to achieve cure differs between individuals and relapse can occur. We asked whether cellular and transcriptional profiling of peripheral blood collected during treatment could identify biomarkers predictive of treatment outcome.

**Methods:**

We analyzed peripheral blood collected during treatment of genotype 1 HCV with 24 weeks of sofosbuvir and weight-based or low dose ribavirin in a trial in which 29% of patients relapsed. Changes in host immunity during treatment were assessed by flow cytometry and whole blood gene expression profiling. Differences in expression of immune-relevant transcripts based on treatment outcome were analyzed using the Nanostring Human Immunology V2 panel.

**Results:**

Multiple cellular populations changed during treatment, but pre-treatment neutrophil counts were lower and natural post-treatment killer cell counts were higher in patients who relapsed. Pre-treatment expression of genes associated with interferon-signaling, T-cell dysfunction, and T-cell co-stimulation differed by treatment outcome. We identified a pre- and post-treatment gene expression signature with high predictive capacity for distinguishing treatment outcome, but neither signature was sufficiently robust to suggest viability for clinical use.

**Conclusions:**

Patients who relapse after hepatitis C virus therapy differ immunologically from non-relapsers based on expression of transcripts related to interferon signaling and T-cell dysfunction, as well as by peripheral neutrophil and NK-cell concentrations. These data provide insight into the host immunologic basis of relapse after DAA therapy for HCV and suggests mechanisms which may be relevant for understanding outcomes with currently approved regimens.

## Background

Over 90% of patients with chronic hepatitis C virus (HCV) infection achieve a sustained virologic response (SVR) after 8–12 weeks of treatment with currently approved direct acting antivirals (DAAs). When 4–6 week courses of treatment are given, treatment failures are unacceptably high. Such failures could relate to drug potency, drug susceptibility of the virus, virus replication levels, and patient adherence, but host factors may also play a role [1–6].

Although race, gender, liver disease stage, baseline viral load, viral genotype, and concomitant medications have correlated with population odds of achieving SVR in some studies, there are currently no clinically reliable predictors of treatment response [6]. Pre-treatment HCV resistance-associated variants (RAVs) associate with relapse with some regimens [7, 8], but existence of RAVs alone cannot predict outcomes in individuals and other factors must be involved [4, 9]. We and others observed higher expression of functional markers of innate, interferon-related, and adaptive immune function in patients who achieved SVR with DAA therapy relative to patients who relapsed [10–16]. We thus hypothesize there is a role for host immunity in modulating, or at least reflecting, the odds of achieving SVR.

Specifically, our analysis of liver biopsies from HCV-infected patients treated with sofosbuvir and ribavirin identified higher post-treatment expression of interferon-related genes in patients achieving SVR, suggesting induction of endogenous type-I interferon activity during therapy may facilitate SVR [14]. In a separate study of PBMCs from two sofosbuvir-based DAA trials, we observed dynamic changes in the composition of peripheral blood during treatment [16], suggesting altered migration of peripheral immune cells during treatment may have relevance for understanding the role of intrahepatic and systemic immunity in viral clearance.

In this study, we combine these observations and ask whether immune markers in peripheral blood before and after treatment with sofosbuvir and ribavirin differed between patients achieving SVR vs. those who relapsed. Patient adherence to antiviral treatment in this trial did not differ based on treatment outcome, but relapse was common, occurring in 29% of subjects [17]. The regimen of sofosbuvir and ribavirin for the treatment of genotype 1 HCV infection is no longer in clinical use due to unacceptably high rates of relapse relative to currently approved therapies. Because relapse was common in this trial, close analysis of longitudinal samples collected during the trial presented an opportunity to understand the mechanistic underpinnings of virologic relapse, which are likely relevant for understanding why relapse can occur with currently approved regimens, albeit less frequently.

## Methods

### Clinical cohort

Clinical samples collected as part of the SPARE clinical trial (NCT01441180) were used in this study. SPARE was a trial conducted at the National Institute of Allergy and Infectious Diseases (NIAID) in which patients infected with HCV genotype-1 were treated with sofosbuvir and weight-based or low dose ribavirin for 24 weeks, as previously described [14, 17]. This study received approval from the Institutional Review Boards at NIAID and the Medical University of South Carolina and was conducted in concordance with the 1975 Declaration of Helsinki. All patients provided written informed consent, as previously reported with initial publication of the clinical trial result [17], which included permissions to use samples and data collected in the trial for future studies.

### Flow cytometry

Immuno-phenotyping of peripheral blood drawn into EDTA-containing blood collection tubes was performed using a modification of the Centers for Disease Control and Prevention guidelines in a Clinical Laboratory Improvement Act-certified laboratory, as previously described [16]. Cells were stained with combinations of monoclonal antibodies and then lysed with Optilyse-C (Beckman Coulter, Hialeah, FL), washed twice, and re-suspended in 500 μl of phosphate-buffered saline (Cambrex, Walkersville, MD). Samples were analyzed immediately on a Becton Dickinson FacsCanto flow cytometer (BDBiosciences, San Jose, CA). 4-color antibody panels used for cellular identification and enumeration have been previously described [16].

Lymphocytes were identified by side scatter and CD45 staining, with confirmation that less than 5% of cells within the lymphocyte gate expressed CD14 [18]. Absolute concentrations of specific lymphocyte populations (CD3+, CD4+, CD8+, CD4+/8+, CD19, and CD16+/56+ lymphocytes) were calculated using the measured percentage of each cell type within the lymphocyte gate and the absolute lymphocyte concentration [16].

### Gene expression analysis

Whole blood was collected into PaxGene RNA tubes at the time of venipuncture either pre-treatment or at the end of treatment, prior to virologic relapse, and was stored at − 80 °C until processing. Paired blood samples were available from 40 subjects enrolled in the trial, including 24 who achieved SVR and 16 who relapsed. RNA was extracted using the PAXgene Blood miRNA Kit (Qiagen) according to the manufacturer’s instructions. RNA quality was assessed using the Agilent Bioanalyzer. RNA samples had a median RNA integrity number (RIN) of 7.2 and most (91%) had an RIN greater than 6.5.

A Nanostring nCounter was used to directly quantitate expression of 579 immune transcripts (Human Immunology v2 Panel). The lower limit of detection is 20 counts, and as such, counts below 25 were considered to be background signal and counts below 100 were considered to be semi-quantitative. Genes in which 30% or more of samples had a count under 100 were excluded from subsequent analysis. Normalization of gene expression was performed relative to housekeeping genes using the GeNorm algorithm. Of 15 housekeeping genes included on the panel, 11 with the lowest standard deviation after normalization were used (all with SD < 0.30). Data were Z-score transformed and grouped by unsupervised clustering. Mean Square Error analysis identified 4 samples with low data quality, and as such these and their paired samples were excluded from further data analysis, yielding a final dataset of 36 paired samples (*n* = 23 SVR, *n* = 13 relapse). Demographic characteristics of these 36 patients are shown in Table 1. More subjects had advanced liver fibrosis (HAI fibrosis 3–4) in the relapse group (*n* = 8) than the SVR group (*n* = 5). Validation of expression for select genes was performed using quantitative reverse transcriptase-polymerase chain reaction (qRT-PCR), as previously described [14].

**Table 1:** Demographic Profile of Patients Included in Whole Blood Transcriptional Analysis

	SVR (*n* = 23)	Relapse (*n* = 13)	
Ribavirin Dose (Low/High)	9/14	9/4	*p* = 0.04 ^a^
Gender (M/F)	13/10	11/2	*p* = 0.08 ^a^
BMI mean (range)	31 (23–46)	29 (19–43)	*p* = 0.39 ^b^
HAI Fibrosis mean (range)	1.17 (0–3)	2.38 (0–4)	*p* = 0.004 ^b^
Pre-treatment ALT mean (range)	51 (28–141)	58 (31–155)	*p* = 0.88 ^b^

*BMI* body mass index, *HAI* Histologic Activity Index, *ALT* Alanine aminotransferase

^a^statistical analysis by Chi-square test

^b^statistical analysis by unpaired t-test

### Immune cell profiling

Advanced Analysis Immune Cell Type Profiling was performed with nSolver software to predict changes in cell type frequencies based on measured expression of gene sets validated to be cell-type specific (Supplemental Table 1) and was compared to data independently derived by flow cytometry [19].

### Statistical analysis

All statistical analyses on NanoString data were performed on log2 transformed normalized counts. Differential expression analyses were carried out using nSolver4.0 and Advanced Analysis package 2.0 (NanoString ^tm^), following the default analysis pipeline. A machine learning algorithm based on Elastic Net, a regression method that applies both the lasso and ridge penalties, was used to discover gene expression signatures that differentiate relapse and SVR patients both pre- and post-treatment. Curated, log2 transformed expression data was used to generate a linear predictor score = ∑*Xiβi* (*X* = gene X expression level; *β* = coefficient). Cross-validation runs were performed to select the strength of each penalty and for estimation of AUC based on the training data set. Demographic and flow cytometry data were analyzed by Chi-square or t-test methods as indicated.

## Results

**Fig. 1:**
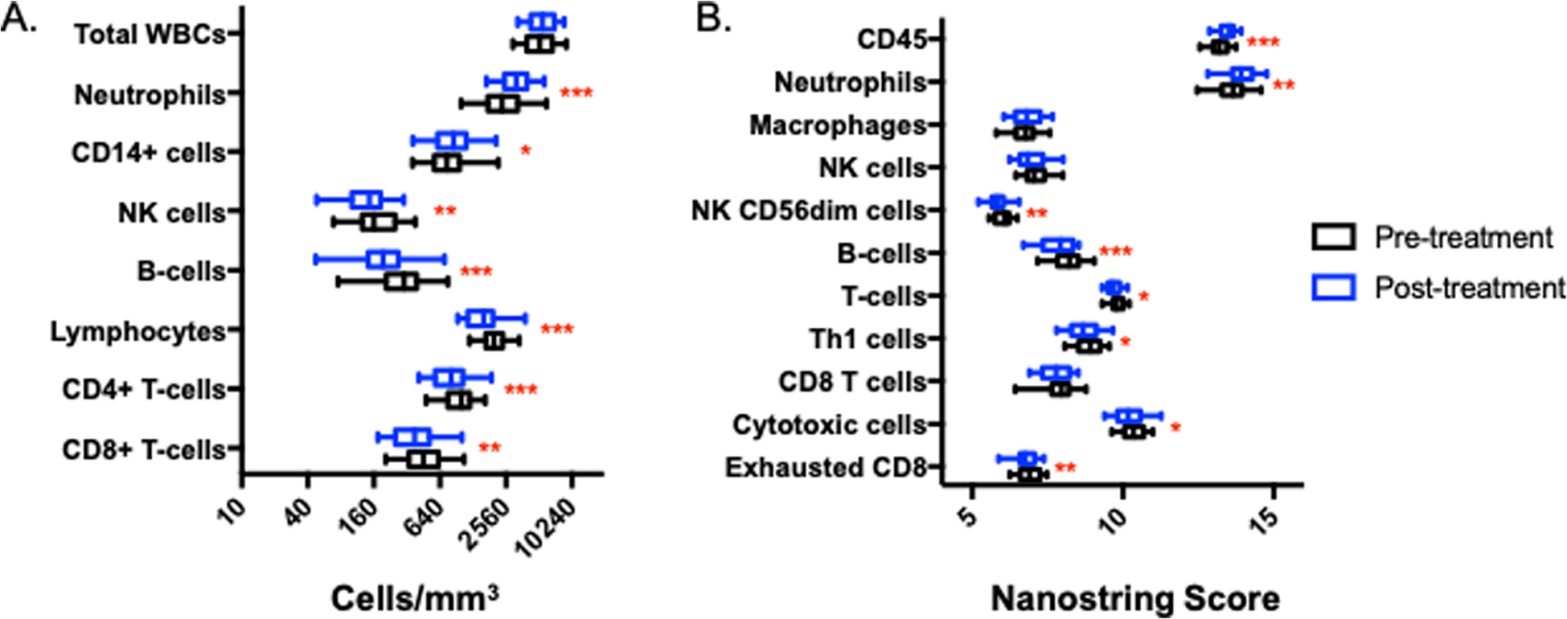
Cellular changes during DAA therapy as measured in peripheral blood by flow cytometry and as predicted by gene expression analysis. **a** Changes in cellular populations determined by flow cytometry, as described in the methods section. Data are derived from up to 53 patients for each individual cell type. **b** Change in predicted cellular composition of total PBMCs using Nanonstring score. Data are derived from 36 patients. Significance was determined by paired t-test with designation on graphs of *p* < 0.05 (*), *p* < 0.005 (**), *p* < 0.0005 (***)

To test the hypothesis that immune differences during DAA treatment associate with treatment outcome, we analyzed peripheral blood from the SPARE trial in which a significant number of patients experienced treatment relapse (29%) [17]. We first examined changes in the cellular composition of peripheral blood over the course of treatment by flow cytometry. We observed a significant decrease in the concentration of total lymphocytes, NK-cells, B-cells, CD4+ T-cells, and CD8+ T-cell during treatment when considering the entire patient cohort (Fig. 1a). We had not observed these declines in data from previously published ribavirin-free DAA cohorts [16], and suspect they may reflect an impact of ribavirin on overall leukocyte counts, as previously reported [20]. We also observed a significant relative increase in neutrophil and CD14+ monocytes cell concentrations over the course of treatment (Fig. 1a).

We next analyzed predicted changes in cellular concentrations calculated by transcriptional analysis of whole blood using the Nanostring Immune Cell Type Profiling platform. Nearly identical results were obtained, as B-cells, NK CD56-dim cells, total T-cells, Th1 cells, cytotoxic CD8+ T-cells, and exhausted CD8+ cells were all predicted to decrease over the course of treatment, while CD45+ cells (a leukocyte marker) and neutrophils were predicted to increase (Fig. 1b). The similarity of findings derived by different techniques suggests whole blood transcriptional profiling was a valid approach to provide meaningful and biologically relevant insight.

**Fig. 2:**
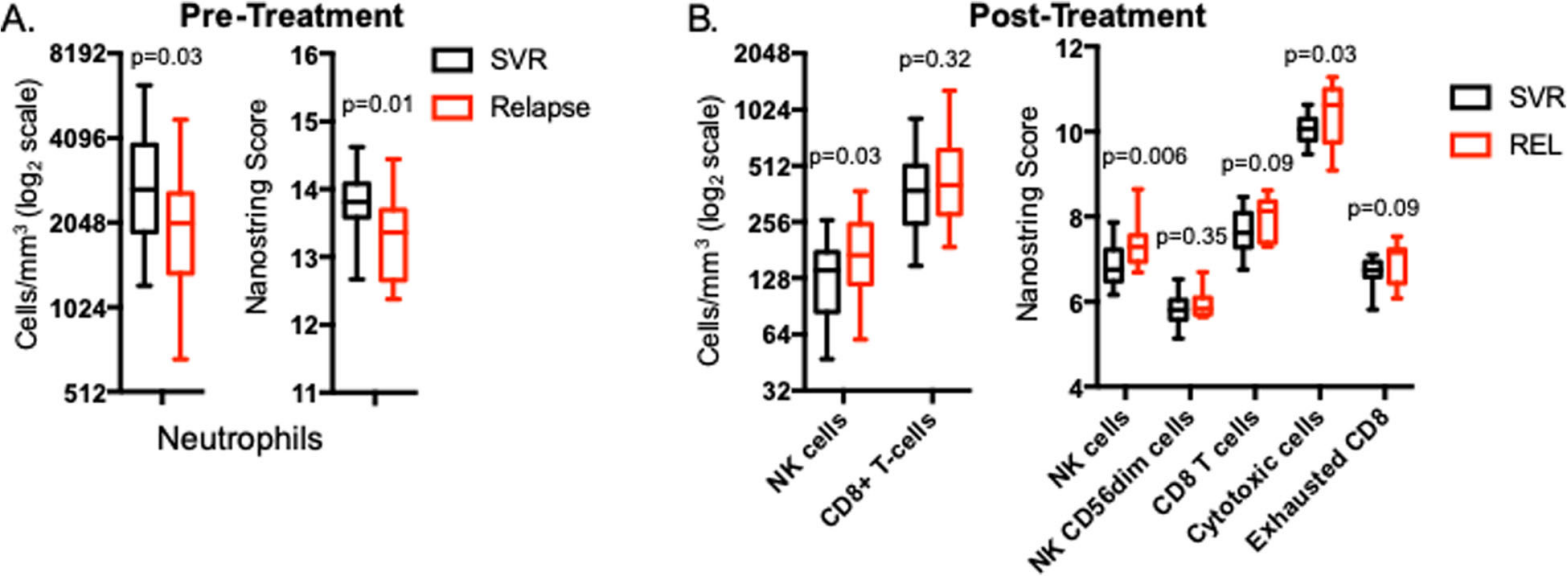
Significant differences in cell type frequency between patients achieving SVR versus patients who relapsed. **a** Pre-treatment, only neutrophil count differed based on eventual treatment outcome, and was found to be lower in relapsers. **b** Post- treatment changes of significance as determined by flow cytometry or Nanostring score. Statistical analysis was by unpaired t-test, with *p*-values displayed

We next examined whether cellular populations differed pre- or post-treatment based on outcome. Pre-treatment, only neutrophil concentration differed by outcome, with relapsers having lower neutrophil concentration as assessed by both flow cytometry and gene expression profiling (Fig. 2a). Post-treatment, relapsers had higher concentrations of NK-cells by flow cytometry (Fig. 2b), but no other significant differences (data not shown). By gene expression profiling, relapsers had higher predicted concentrations of NK cells and cytotoxic cells, and a trend towards higher concentrations of total CD8+ T-cells and exhausted CD8+ T-cells (Fig. 2b).

**Fig. 3:**
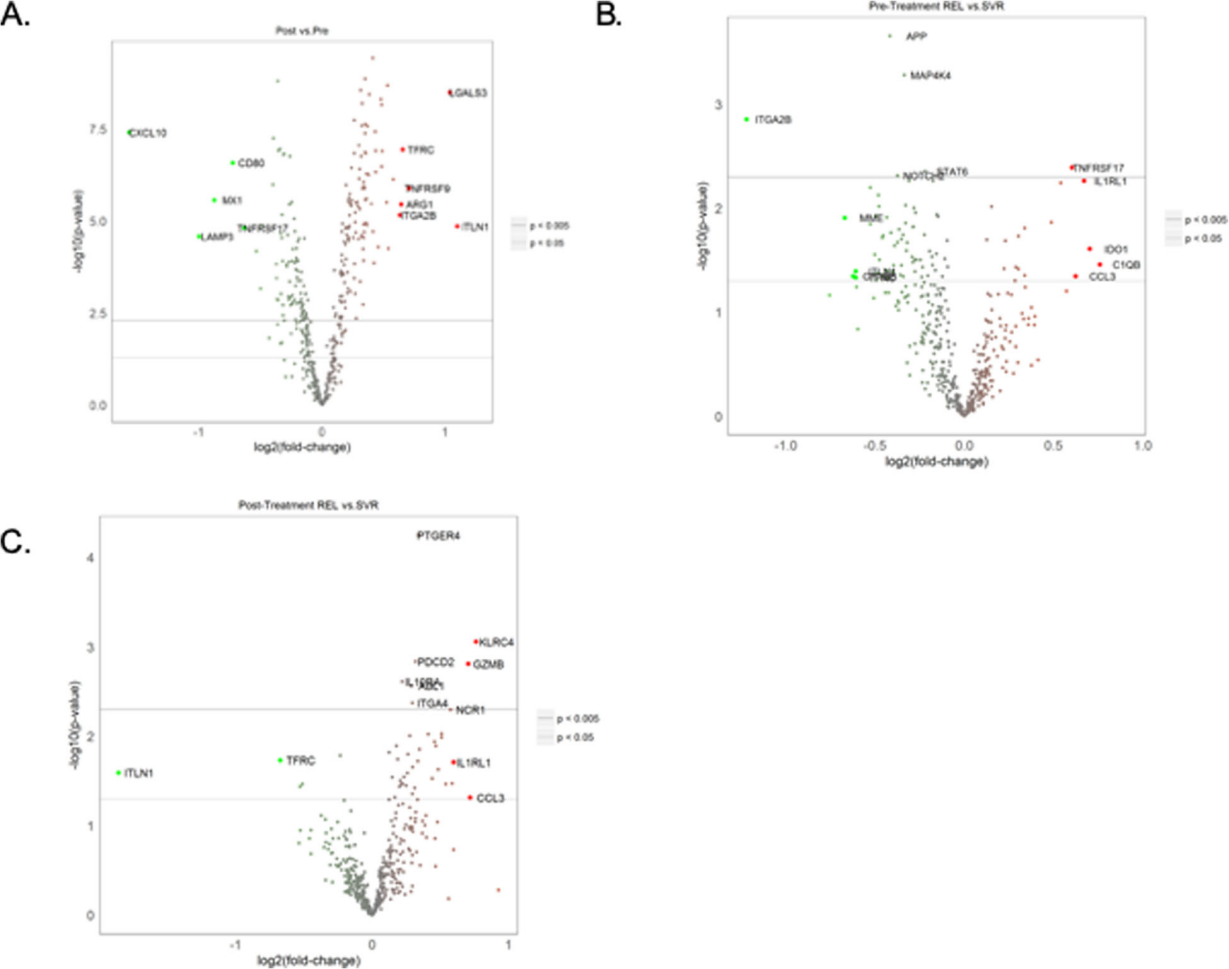
Volcano plot for genes that change over time in the entire patient cohort (**a**), or that have differential expression pre- (**b**) or post-treatment (**c**) based on treatment response. For A, genes in green with a negative log fold change had decreased expression during treatment, while genes in red with a positive fold change had increased expression. For B-C, genes with a negative fold change (in green) had lower expression in relapsers compared to patients achieving SVR, while genes with a positive fold change (in red) had higher expression in relapsers. Genes with labels have a *p-*value < 0.05 and a fold change of 1.5 or greater

We next analyzed expression of individual transcripts and examined changes with treatment and differences by outcome. In the entire patient cohort, 208 genes changed significantly during treatment (Fig. 3a, Supplemental Excel File Tab 1), a substantial percentage of the total genes tested. Multiple ISGs decreased with treatment (e.g. *MX1*, *IFIT2, CXCL10, RARRES3*), consistent with prior results from other DAA trials [14, 21, 22]. *IFNAR1* and *IFNAR2*, which comprise the type-I interferon receptor, both had increased expression post-treatment. Genes associated with T-cell activation (e.g. *CD80*, *CD160*, *LAG3*, *BTLA*, *ICOS*) decreased with treatment. An analysis of the data by hierarchical clustering to examine any impact of gender, IFNL4 genotype, fibrosis stage, ribavirin dose, and pre-treatment ALT levels, several of which differed based on outcome in this patient cohort (Table 1), did not identify clear clustering based on these factors (Supplemental Fig. 1).

**Fig. 4:**
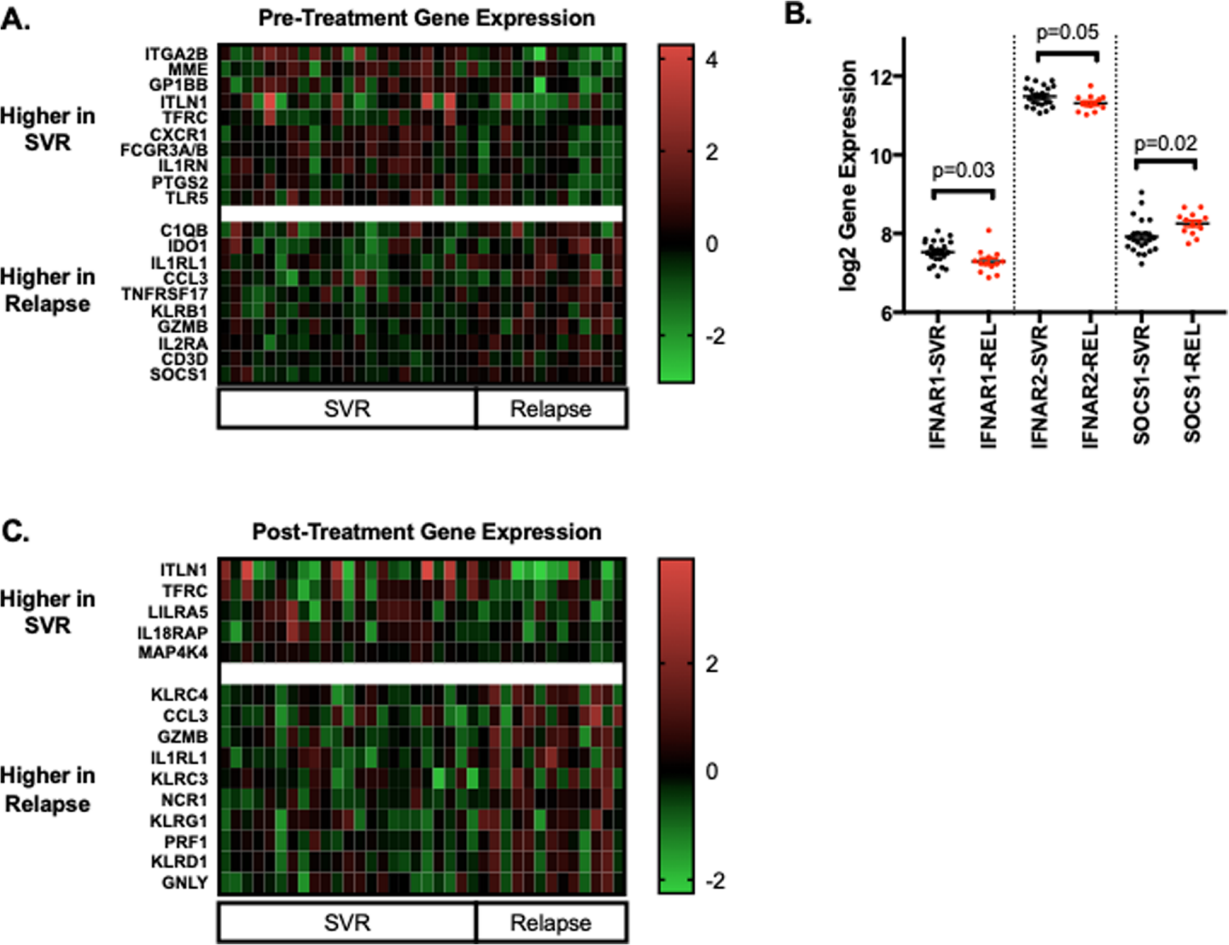
Genes with the highest differential expression between SVR and relapse in whole blood either pre- (**a-b**) or post- treatment (**c**). Delta log2 gene expression was determined for each patient in relation the mean expression for the cohort and all displayed genes achieved significance. *P*-values for (**b**) were determined by unpaired t-test

We next analyzed differences by treatment outcome and identified 84 genes pre-treatment and 43 genes post-treatment with differential expression (Fig. 3b-c, Supplemental Excel File Tabs 2–3). Significant genes with the highest fold differential expression between SVR and relapse patients pre- and post-treatment are displayed as heatmaps (Fig. 4).

Pre-treatment, prior to DAA exposure, a number of genes related to TLR-mediated pathogen recognition (*TLR8, TLR2, TLR5, IRAK4, TOLLIP)*, IL1 signaling, complement activation, and TNF signaling had lower expression in patients who later experienced treatment relapse. Multiple genes related to neutrophils (*FCGR3A/B, FCGR2A, FCGR2A/C*) were lower in relapsers, consistent with observations derived by flow cytometry. Interestingly, pre-treatment expression of *IFNAR1*, *IFNAR2,* and *SOCS3* was lower in relapsers, while pre-treatment expression of *SOCS1* was higher (Fig. 4a and Supplemental Excel File Tab. 2). *SOCS3* and *SOCS1* are known negative regulators of interferon signaling. Several genes associated with T-cell dysfunction/tolerance (*CTLA4-TM, CD244, IDO1*) had higher pre-treatment in patients who subsequently relapsed.

Post-treatment, several consistent changes distinguished the groups by outcome. First, patients who relapsed had higher expression of genes related to NK-cell frequency and function (*KLRK1, KLRD1, KLRG1, NCR1, KLRC3, KLRC4, PRF1, GZMB)*, which includes both activating and inhibitory receptors, consistent with data derived by flow cytometry. In addition, patients who relapsed had higher expression of genes associated with T-cell dysfunction (*HAVCR2, KLRG1, CD244*).

Finally, we asked whether expression of any gene or combination of genes pre-or post-treatment could have sufficient capacity to robustly predict clinical outcome. Using a machine learning algorithm, we identified a 12-gene pre-treatment and an 5-gene post-treatment set with high predictive capacity in training sets with AUCs of 0.95 and 0.86, respectively (Supplemental Figs. 2–3). As we lacked data from an independent cohort to validate these gene signatures, we performed an internal validation analysis and found neither signature had a consistently high AUC on cross-validation runs, both with mean AUCs of 0.66 (Supplemental Figs. 2–3).

## Discussion

This study asked whether analysis of routinely available clinical samples could distinguish HCV patients who relapse or achieve SVR after treatment with sofosbuvir and ribavirin and provide insight into mechanisms of relapse. While the antiviral regimen of sofosbuvir and ribavirin is no longer in routine clinical use, the high rates of relapse that occurred in the study offered an opportunity to identify biologic correlates of relapse that likely remain relevant when trying to understand mechanisms of relapse that occur with currently approved DAA regimens. We identified differences in neutrophil count pre-treatment and NK cell count post-treatment that distinguish patients by outcome. In addition, whole blood expression of transcripts related to interferon signaling, T-cell dysfunction, and NK-cells distinguished patients by treatment outcome. These data provide evidence that heightened IFN signaling and dysregulated adaptive immunity pre-treatment associate with higher odds of relapse.

Pre-treatment, our primary observation was that neutrophil count was lower in eventual relapsers and that expression of genes related to pathogen detection, neutrophil function, and interferon signaling differed by outcome. While multiple canonical ISGs decreased during treatment and did not differ by treatment outcome (e.g. *CXCL10, RARRES3, MX1*), we were intrigued to find that expression of both *IFNAR1* and *IFNAR2* increased with treatment, suggesting an inverse correlation between type-I receptor expression level and interferon signaling. These data are consistent with previous reports that *IFNAR1* gene expression is reduced in PBMC of chronic HCV patients [23]. Our observation that patients who relapsed had lower baseline expression of both *IFNAR1* and *IFNAR2* thus suggests a heightened state of interferon signaling pre-treatment. This observation suggests that, as was trued for interferon-based therapy [24], heightened interferon signaling pre-treatment may reflect a poor antiviral immune state less amenable to HCV clearance, and thus more prone to relapse.

Although type-I and type-III IFN signaling induce a similar ISG transcriptional profile in cells expressing both receptors, the pattern of genes is not identical and the kinetics of induction can differ [25]. As noted above, the expression of *SOCS1*, a negative regulator of IFN signaling, decreased during treatment, consistent with the pattern observed for other ISGs. Expression was higher pre-treatment in relapsers, which was the inverse pattern of that observed with *IFNAR1* and *IFNAR2*. This data is intriguing as type-III IFN signaling has previously been shown to selectively induce SOCS1 relative to type-I IFN signaling in human and chimpanzee liver [26, 27], suggesting the chronic IFN signature in HCV patients could relate to type-III IFN signaling. Whether the lower neutrophil count we observed pre-treatment in relapsers reflects higher levels of endogenous interferon signaling, as interferon treatment causes a relative neutropenia [28, 29], requires further study. This observation regarding neutrophils, which contribute significantly to the cellular composition of whole blood analyzed by gene expression profiling, is of particular interest as neutrophils are excluded from most analyses examining PBMCs. Taken together, these data suggest higher baseline IFN activation in patients who subsequently relapsed.

Post-treatment, relapsers had higher levels of NK cells and NK-cell associated transcripts than patients achieving SVR. This is intriguing as changes in NK cell function and frequency during DAA therapy have been previously reported, with most observing decreases during HCV treatment, including reduced surface expression of NKp30 (NCR3), NKp46 (NCR1), TRAIL, and NKG2A [10, 30]. Our data suggests NK-cell frequency and relative activation state post-treatment could reflect presence of residual intrahepatic HCV. While NKp30 and NKG2A were not analyzed in this study, we found no change in TRAIL or NKp46 expression during treatment, but did find higher expression of the activating receptor NKp46 post-treatment in relapsers. Our data are also intriguing in the light that IFNL genotype and IFN activity have been linked to differential NK cell activation [31–34]. As understanding NK-cell functional status from whole blood gene expression profiling is challenging, our data support future experiments focused on analysis of NK-cell phenotype at the end of treatment.

Another major finding of the study relates to genes associated with T-cell dysfunction. We observed significantly higher post-treatment expression of markers of T-cell exhaustion in relapsers including *HAVCR2* (*TIM3*) and *KLRG1* (Fig. 4) [35] .While *CTLA-4-TM* decreased over the course of treatment in the entire cohort, relapsers had higher pre-treatment expression. In addition, while 2B4 (*CD244*) did not change during treatment, patients who relapsed had higher expression both pre- and post-treatment. Taken together, these data associate relapse with T-cell dysfunction, consistent with prior findings derived by flow cytometry [10–15]. Prior work identified a heterogenous response to in vitro blockade of inhibitory co-stimulatory receptors (PD1, CTLA4, TIM3, 2B4) for the CD8 response in patients with HCV infection [36], and may reflect distinct ways in which individual hosts respond to the same pathogen, which could have relevance for treatment relapse. In our study we observed no change in *PDL1* (*CD274*) during treatment and no difference pre- or post-treatment based on treatment outcome, while expression of *PD1* (*PDCD1)* was low and did not satisfy the stringency criteria of this analysis. As IFN-driven signaling can cause T-cell exhaustion, which can be reversed with IFN blockade [37], our data suggesting heightened interferon signaling pre-treatment in relapsers may thus relate mechanistically to parallel observations of higher T-cell dysfunction in relapsers.

Together, these data suggest relapsers might be more likely to respond if they could be identified prospectively, and if the rate of their immune restoration on DAA therapy could be identified and considered when considering treatment duration. Given the high efficacy of currently approved DAA regimens, it is intriguing to consider that subjects experiencing more rapid immune “rejuvenation” could achieve cure with shortened treatment durations, while those with a more sluggish immune response on therapy may benefit from a longer course of treatment to minimize the risk of relapse. When considering other regulators of T-cell activation [38], we did observe down-regulation of *CD80*, *CD160*, *LAG3*, *BTLA*, and *ICOS* with treatment (Fig. 4, Supplemental Excel File Tab 1), but saw no difference at any time point based on treatment outcome.

A limitation of our study is that most transcripts are not cell-type specific. As such our suggestions regarding mechanism and causation require prospective validation. Other limitations include the low number of patients with IFNL4 genotype predicting inability to produce IFNL4 protein, limiting our capacity to analyze difference in gene expression patterns attributable to this. Although more patients with advanced fibrosis were present in the group of patients who relapsed (Table 1), which could have confounded our analysis, we did not identify an association of fibrosis with the gene expression patterns that correlated with relapse in this study. While we did not identify an impact of ribavirin dose on the reported results, because ribavirin can impact gene expression and impart sensitization to IFN signaling [39] an impact of ribavirin dose on these findings cannot be ruled out [17]. Finally, as discussed above, sofosbuvir and ribavirin is not currently in clinical use for treatment of genotype-1 HCV infection due to high rates of relapse. Nonetheless, the biologic correlates identified in this study likely have relevance for understanding why relapse still occurs in a subset of subjects receiving current regimens, and as such these findings will benefit from further validation using samples from cohorts receiving ribavirin-free regimens.

## Conclusions

In conclusion, neutrophil count pre-treatment and NK-cell count post-treatment distinguish patients achieving SVR from those who relapse after sofosbuvir and ribavirin treatment. In addition, expression of interferon related genes and genes related to T-cell dysfunction differed by outcome, supporting a role for host immunity in modulating HCV treatment outcome with DAA therapy. These findings suggest that favorable host immunity could enable higher response rates and shorter durations of therapy, which could have significant economic and adherence implications.

## Supplementary Information

**Additional file 1: Supplemental Fig. 1**: Overview of raw gene expression data. Hierarchical clustering of samples based on similarity of global gene expression. Shown are potentially clinically relevant variables including treatment response (SVR vs REL), gender (male, female), ribavirin dose (weight-based versus low dose ribavirin), fibrosis score (HAI fibrosis), IFNL4 genotype (rs368234815 locus where 2 = ΔG allele and 1 = T allele), and ALT level. Whether samples were pre or post-treatment is shown on the bottom x-axis.

**Additional file 2: Supplemental Fig. 2**: Predictive capacity of genes expressed pre-treatment. Shown is a heatmap of genes identified by machine learning algorithm to best associate with outcome, performance of the training gene set by predictive score and AUC, and performance of the gene set when the data were re-analyzed with by cross-validation runs.

**Additional file 3: Supplemental Fig. 3**: Predictive capacity of genes expressed post-treatment. Shown is a heatmap of genes identified by machine learning algorithm to best associate with outcome, performance of the training gene set by predictive score and AUC, and performance of the gene set when the data were re-analyzed with by cross-validation runs.

**Additional file 4: Supplemental Table 1**: Genes on Nanostring Immunology panel used to assess cell-type frequency in whole blood.

**Additional file 5.**

## Data Availability

The datasets used and/or analyzed during the current study are available from the corresponding author on reasonable request.

## Abbreviations

DAA: Direct acting antiviral
HCV: Hepatitis C virus
NIAID: National Institute of Allergy and Infectious Diseases
NK-cell: Natural Killer cell
PBMCs: Peripheral blood mononuclear cells
qRT-PCR: Quantitative reverse transcriptase-polymerase chain reaction
RAV: Resistance associated variant
SVR: Sustained Virologic Response
