# Supplementary figures and images for: Peripheral blood correlates of virologic relapse after Sofosbuvir and Ribavirin treatment of Genotype-1 HCV infection
Source: BMC Infect Dis. 2020 Dec 4;20:929. doi: 10.1186/s12879-020-05657-5 (PMC7718661; doi:10.1186/s12879-020-05657-5)

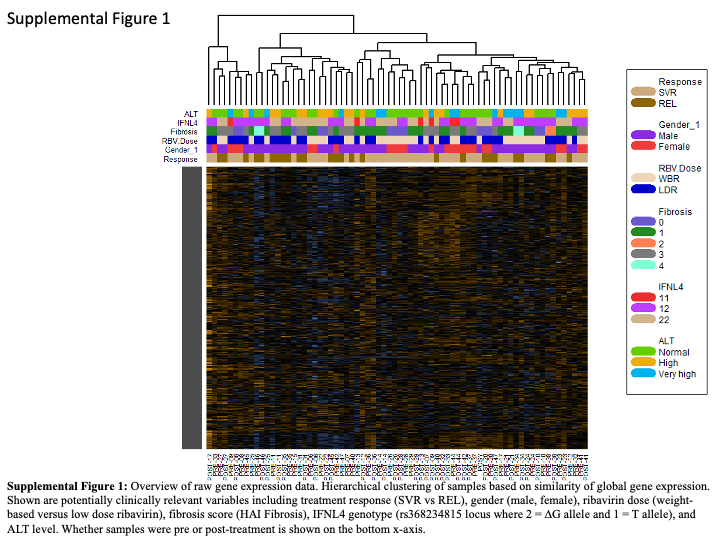

Supplement: Supplementary file 1 — Additional file 1: Supplemental Fig. 1: Overview of raw gene expression data. Hierarchical clustering of samples based on similarity of global gene expression. Shown are potentially clinically relevant variables including treatment response (SVR vs REL), gender (male, female), ribavirin dose (weight-based versus low dose ribavirin), fibrosis score (HAI fibrosis), IFNL4 genotype (rs368234815 locus where 2 = ΔG allele and 1 = T allele), and ALT level. Whether samples were pre or post-treatment is shown on the bottom x-axis. [file 12879_2020_5657_MOESM1_ESM.tiff]

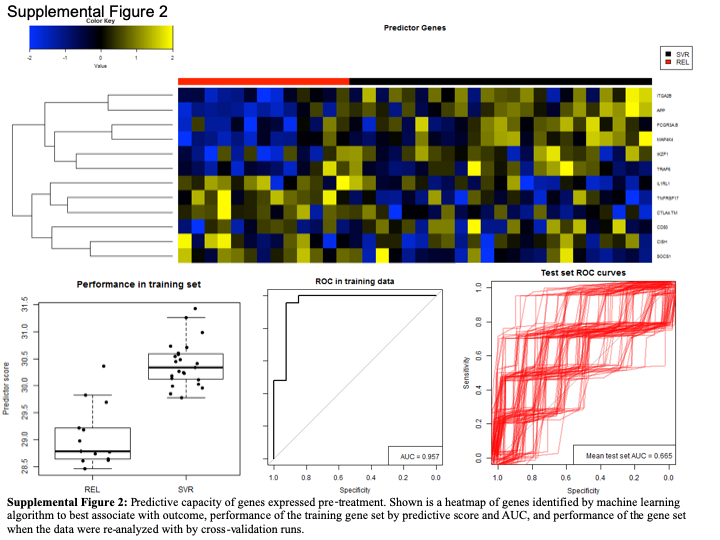

Supplement: Supplementary file 2 — Additional file 2: Supplemental Fig. 2: Predictive capacity of genes expressed pre-treatment. Shown is a heatmap of genes identified by machine learning algorithm to best associate with outcome, performance of the training gene set by predictive score and AUC, and performance of the gene set when the data were re-analyzed with by cross-validation runs. [file 12879_2020_5657_MOESM2_ESM.tiff]

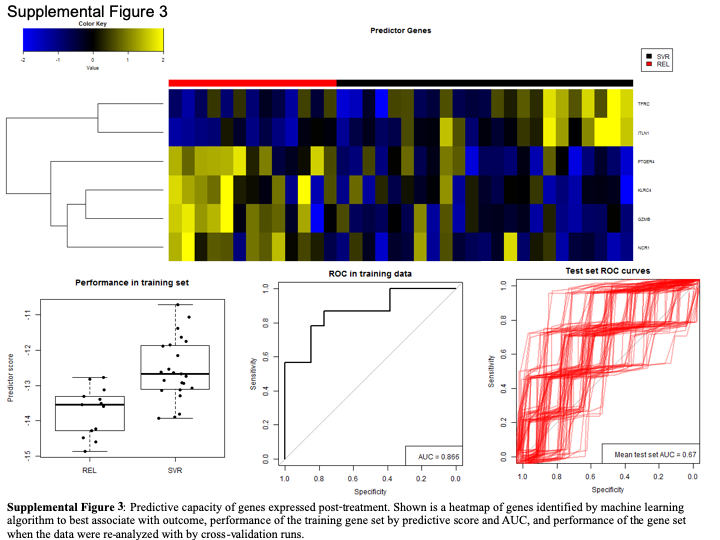

Supplement: Supplementary file 3 — Additional file 3: Supplemental Fig. 3: Predictive capacity of genes expressed post-treatment. Shown is a heatmap of genes identified by machine learning algorithm to best associate with outcome, performance of the training gene set by predictive score and AUC, and performance of the gene set when the data were re-analyzed with by cross-validation runs. [file 12879_2020_5657_MOESM3_ESM.tiff]
