# Supplementary material for: Peripheral blood correlates of virologic relapse after Sofosbuvir and Ribavirin treatment of Genotype-1 HCV infection
Source: BMC Infect Dis. 2020 Dec 4;20:929. doi: 10.1186/s12879-020-05657-5 (PMC7718661; doi:10.1186/s12879-020-05657-5)
Supplement: Supplementary file 4 — Additional file 4: Supplemental Table 1: Genes on Nanostring Immunology panel used to assess cell-type frequency in whole blood. [file 12879_2020_5657_MOESM4_ESM.docx]

**Supplemental Table 1**: Genes on Nanostring Immunology panel used to assess cell-type frequency in whole blood.

| Cell Type | Selected Marker Genes |
| --- | --- |
| B-cells | BLK, CD19, FCRL2, MS4A1, KIAA0125, TNFRSF17, TCL1A, SPIB, PNOC |
| CD45 | PTRPC |
| Cytotoxic cells | PRF1, GZMA, GZMB, NKG7, GZMH, KLRK1, KLRB1, KLRD1, CTSW, GNLY |
| DC | CCL13, CD244, EOMES, PTGER4 |
| Exhausted CD8 | LAG3, CD244, EOMES, PTGER4 |
| Macrophages | CD68, CD84, CD163, MS4A4A |
| Mast cells | TPSB2, TPSAB1, CPA3, MS4A2, HDC |
| Neutrophils | FPR1, SIGLEC5, CSF3R, FCAR, FCGR3B, CEACAM3, S100A12 |
| NK CD56dim cells | KIR2DL3, KIR3DL1, KIR3DL2, IL21R |
| NK cells | XCL1, XCL2, NCR1 |
| T-cells | CD6, CD3D, CD3E, SH2D1A, TRAT1, CD3G |
| Th1 cells | TBX21 |
| Treg | FOXP3 |
| CD8 T cells | CD8A, CD8B |
